# HapKled: a haplotype-aware structural variant calling approach for Oxford nanopore sequencing data

**DOI:** 10.3389/fgene.2024.1435087

**Published:** 2024-07-09

**Authors:** Zhendong Zhang, Yue Liu, Xin Li, Yadong Liu, Yadong Wang, Tao Jiang

**Affiliations:** ^1^ Faculty of Computing, Harbin Institute of Technology, Harbin, Heilongjiang, China; ^2^ Zhengzhou Research Institute, Harbin Institute of Technology, Zhengzhou, Henan, China

**Keywords:** structural variant, variant calling, haplotype-tagging, Oxford nanopore sequencing, long-read sequencing

## Abstract

**Introduction:** Structural Variants (SVs) are a type of variation that can significantly influence phenotypes and cause diseases. Thus, the accurate detection of SVs is a vital part of modern genetic analysis. The advent of long-read sequencing technology ushers in a new era of more accurate and comprehensive SV calling, and many tools have been developed to call SVs using long-read data. Haplotype-tagging is a procedure that can tag haplotype information on reads and can thus potentially improve the SV detection; nevertheless, few methods make use of this information. In this article, we introduce HapKled, a new SV detection tool that can accurately detect SVs from Oxford Nanopore Technologies (ONT) long-read alignment data.

**Methods:** HapKled utilizes haplotype information underlying alignment data by conducting haplotype-tagging using Whatshap on the reads to improve the detection performance, with three unique calling mechanics including altering clustering conditions according to haplotype information of signatures, determination of similar SVs based on haplotype information, and slack filtering conditions based on haplotype quality.

**Results:** In our evaluations, HapKled outperformed state-of-the-art tools and can deliver better SV detection results on both simulated and real sequencing data. The code and experiments of HapKled can be obtained from https://github.com/CoREse/HapKled.

**Discussion:** With the superb SV detection performance that HapKled can deliver, HapKled could be useful in bioinformatics research, clinical diagnosis, and medical research and development.

## 1 Introduction

Variants are a type of genetic mechanism that greatly influence the phenotypes of humans and can cause many genetic diseases. Thus, accurate detection of variants bears great significance to genetic research, clinical diagnosis, and medical research (Kim and Misra, 2007; Conrad et al., 2010; Auton et al., 2015; Chiang et al., 2017; Bennett et al., 2020). Among all types of variants, i.e., single-nucleotide variants (SNVs), small insertions/deletions (indels), and structural variants (SVs), SVs are variants that influence genetic areas of no less than 50 base pairs (bp) (Kidd et al., 2010; Sudmant et al., 2015; Chiang et al., 2017; Ahsan et al., 2023) and have a more significant influence on the phenotypes and diseases due to their large sizes and largest influenced genomic areas (Weischenfeldt et al., 2013; Macintyre et al., 2016; Chiang et al., 2017; Dennenmoser et al., 2017; Jeffares et al., 2017). Furthermore, the large size and varied types (typically including deletions, insertions, duplications, and inversions) of SVs make them harder to discover than SNVs and indels (Kosugi et al., 2019; Kosugi and Terao, 2024). As a result, the detection of SVs is both important and challenging.

The advent of next-generation sequencing (NGS) has made rapid and affordable detection of SVs possible (Hu et al., 2021), and many tools (Layer et al., 2014; Chen et al., 2016; Li et al., 2021; Liu et al., 2021; Zhang et al., 2021) have been developed to detect indels or SVs on NGS data. However, due to the limited read length, detecting SVs on NGS data remains a formidable task, especially for those SVs longer than the read length of NGS data (English et al., 2015). The long-read sequencing technologies, including Pacific Biosciences (PacBio) and Oxford Nanopore Technologies (ONT), have partially solved this problem due to their significantly longer read lengths (Roberts et al., 2013; Jain et al., 2016). The longer read length makes it more likely that the SVs are included in a single read, thus leaving intact signatures within the read that can be more easily identified by SV detection tools, and also makes reads that include large altered areas caused by SVs to be more easily mapped to the reference genome (Goodwin et al., 2016; Sedlazeck et al., 2018a). Many tools have been created to detect SVs on long-read data, including kled (Zhang et al., 2024), cuteSV (Jiang et al., 2020), and Sniffles (Sedlazeck et al., 2018b; Smolka et al., 2024), among others (Heller and Vingron, 2019; Jiang et al., 2019a, 2019b).

Although these SV detecting tools can quickly and accurately detect SVs from long-read alignment data, there is still room for improvements. Currently, there are several haplotype-tagging tools available (Martin et al., 2016; Fu et al., 2023). These tools can haplotype-tag alignment files and output haplotype-tagged reads, providing haplotype information to SV calling tools, potentially resulting in more precise and comprehensive SV detection. Using the haplotype information of reads, we can improve the results of SV calling in the following aspects: when clustering signatures extracted from reads, the haplotype information can be used to either cluster the signatures from each haplotype separately or apply different conditions for signatures from the same haplotype or different haplotypes; before reporting the results, we can use the haplotype information of the cluster to improve the filtering process. Duet (Zhou et al., 2022) is an SV calling tool that first haplotype-tags the reads, then calls SVs using cuteSV, and subsequently uses haplotype information generated using Clair3 (Zheng et al., 2022) and WhatsHap to phase and filter the SVs.

In this article, we introduce HapKled, an SV detection tool that accurately detects SVs on ONT sequencing data based on haplotype-aware strategies. Unlike traditional SV detection tools, HapKled first haplotype-tags the reads in the input alignment file and then detects SVs from these haplotype-tagged alignments utilizing three innovative features: applying different conditions for signatures from the same or different haplotypes when clustering, distinguishing between similar and near SVs from different haplotypes, and adjusting the filtering parameters according to different haplotype-tagging qualities. During rigorous experiments, HapKled demonstrated superior performance compared to state-of-the-art SV detection tools. We believe that, with the excellent SV detection performance delivered by HapKled, it could be useful in bioinformatics research, clinical diagnosis, and medical research and development.

## 2 Materials and methods

**FIGURE 1:**
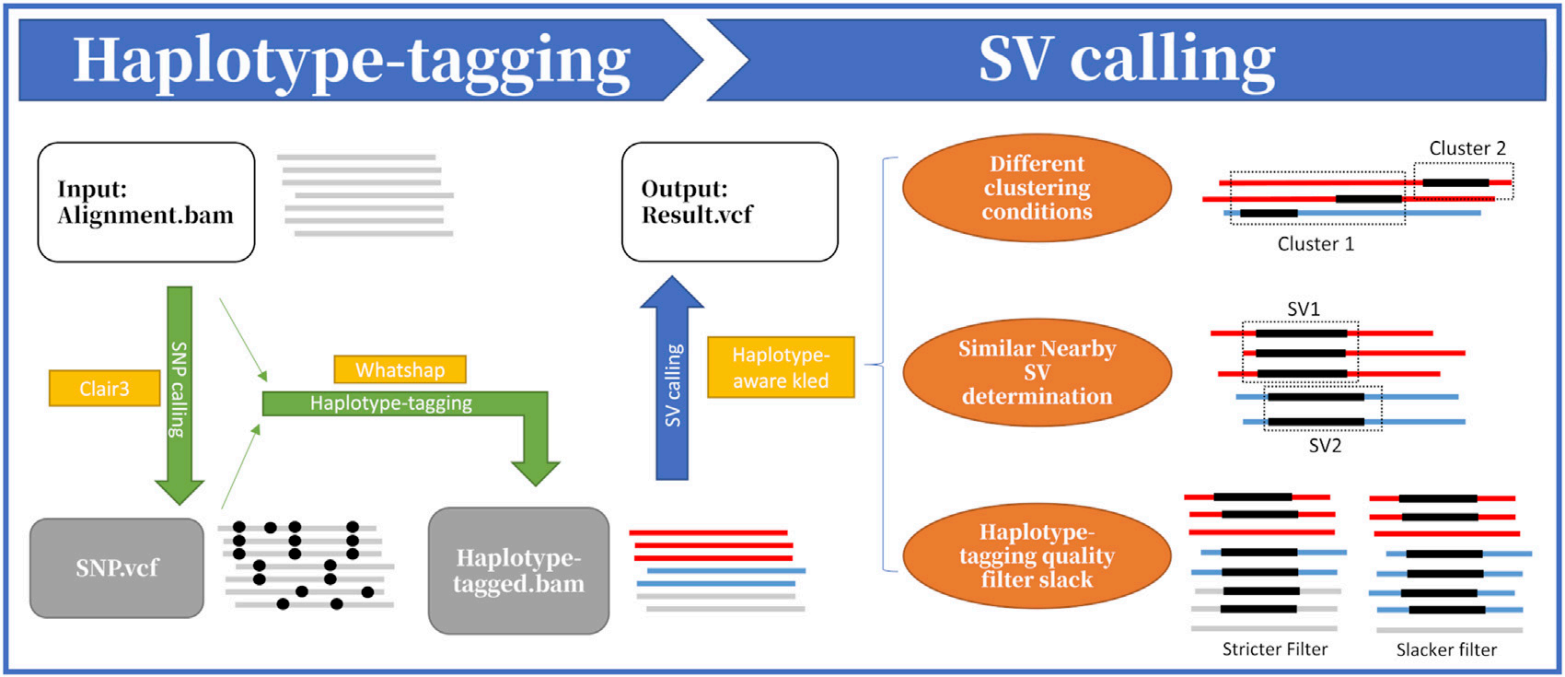
Overview of HapKled procedures. Part 1: the input alignment file is first used to call SNVs using Clair3, and then HapKled uses the detection result to haplotype-tag the alignment file using WhatsHap. Part 2: with the haplotype-tagged reads generated in Part 1, HapKled uses a haplotype-aware version of kled with three improvements, i.e., applying different conditions when clustering, distinguishing similar nearby SVs based on per-haplotype statistics, and adjusting filtering parameters based on haplotype-tagging quality, to generate the final VCF.

As shown in Figure 1, HapKled takes an alignment BAM file as input and outputs a variant call format (VCF) file containing the detected SVs. The procedures mainly contain two parts: the haplotype-tagging part and the SV calling part. In the haplotype-tagging part, HapKled takes the alignment BAM file as input, uses Clair3 and WhatsHap to add haplotype information to the reads, and generates a new BAM file that contains the reads with haplotype information; and in the following SV calling part, HapKled utilizes a haplotype-aware kled to call SVs from the haplotype-tagged BAM file and outputs the final VCF file.

### 2.1 Haplotype-tagging

The procedures of haplotype-tagging consist of two steps: SNV calling using Clair3 (v1.0.5), and haplotype-tagging using WhatsHap (v1.7). HapKled first uses Clair3 to call SNVs from the input alignment file with parameters platform = “ont” --model_path = “r941_prom_hac_g360 + *g*422” and generates a VCF file containing the SNVs. After that, HapKled uses WhatsHap to haplotype-tag the input alignment file by utilizing the SNV information with parameters “--ignore-read-groups--indels,” and generates a BAM file that contains the haplotype-tagged reads, which are used in the haplotype-aware SV calling.

### 2.2 Haplotype-aware kled

The SV calling part of HapKled takes the haplotype-tagged BAM file generated in the haplotype-tagging part and uses a modified version of kled, which is haplotype-aware, with three unique improvements.

#### 2.2.1 Different clustering conditions

When clustering, as in the original kled, HapKled first clusters every SV signature extracted from reads and merges any two clusters if there are two signatures from these two clusters that are similar enough. Formally, for clusters $C_{1}$ and $C_{2}$ , if there exist $S_{1}\in C_{1}$ and $S_{2}\in C_{2}$ , where any condition in Eqs 1, 2 is satisfied, HapKled merges $C_{1}$ and $C_{2}$ . Here, ${S_{i}}_{Left}$ **,** ${S_{i}}_{Right},$ and ${S_{i}}_{Length}$ represent the leftmost position, rightmost position, and length of $S_{i}$ , i=1,2. F and CR are predefined SV type specific parameters, respectively.

$$\max\left(\left|{S_{1}}_{Left}-{S_{2}}_{Left}\right|,\left|{S_{1}}_{Right}-{S_{2}}_{Right}\right|\right)<F,$$ (1)

$$\frac{\max\left(\left|{S_{1}}_{Left}-{S_{2}}_{Left}\right|,\left|{S_{1}}_{Right}-{S_{2}}_{Right}\right|,\left|{S_{1}}_{Length}-{S_{2}}_{Length}\right|\right)}{\min\;\left({S_{1}}_{Length},{S_{2}}_{Length}\right)}<CR.$$ (2)

HapKled further improves this procedure when haplotype information is available: if two signatures are from the same haplotype, it faces no inter-haplotype interferences; thus, it should have stricter conditions when considering merging and *vice versa*. Formally, when comparing (1) and (2), F and CR are multiplied by SR , if $S_{1}$ and $S_{2}$ are from the same haplotype, or multiplied with DR , if $S_{1}$ and $S_{2}$ are from different haplotypes. $SR\in \left[0,1\right]$ and $\left.DR\in \left[1,+\right.\infty \right)$ are SV type specific parameters.

#### 2.2.2 Similar nearby SV distinction

There are some circumstances in which two nearby SVs reside in different haplotypes, coincidentally having the same SV type and similar SV lengths. In the traditional method of SV detection, these SVs are very likely to be clustered into the same cluster in the clustering procedure because they share similar locations and lengths, and it is hard to distinguish them from each other. However, with haplotype information, we can determine them by the following method: assuming within a certain cluster, the average lengths of SV signatures from haplotype 1 are significantly different from those from haplotype 2, HapKled disunites the cluster into two clusters containing signatures from each haplotype, along with those signatures from reads that have unknown haplotypes. Two lengths are considered significantly different if the condition in (3) is met, where $M_{i}$ and ${SD}_{i}$ , i=1,2 are the mean value and standard deviation of the lengths of the signatures from haplotype 1 and haplotype 2, respectively.

$$\left|M_{1}-M_{2}\right|>\max\left({SD}_{1},{SD}_{2}\right).$$ (3)

#### 2.2.3 Filtering adjustments based on haplotype-tagging quality

After clustering, HapKled refines and filters the clusters to get the final VCF containing called SVs based on the methods of kled. HapKled sets the POS and SVLEN fields in the VCF records as the mean of the positions and lengths of the signatures in the cluster, respectively, and filters out clusters that have fewer supported reads than FF+FR∗Cov and a less consistent score of lengths than FS , where the consistent score is defined as $1-\frac{standard\;deviation\;of\;signature\;lengths}{\max\;length\;of\;signatures}$ ; Cov is the average read depth of the chromosome; and FF,FR , and FS are SV type specific parameters. There are two sets of FF , FR , and FS clusters that fail if both sets are filtered out.

The quality of a cluster can be influenced by many factors: the complexity of this genomic area, sequencing quality, mapping quality, etc. It is natural to think that haplotype-tagging quality can represent the overall quality of the cluster to a certain degree. Thus, we relax the parameters in the filtering step if the haplotype-tagging quality is high. Specifically, if most signatures that constitute the cluster are from successfully haplotype-tagged reads, HapKled considers this cluster as a high haplotype-tagging quality cluster, and if the cluster’s most haplotype-tagged reads come from the same haplotype, we relax the parameters in the refining step accordingly; when the haplotype-tagged reads come from different haplotypes, the circumstance is more complicated: on one hand, this area has a high haplotype-tagged ratio, potentially representing a high sequencing and mapping quality; on the other hand, the signatures of this cluster come from different haplotypes, potentially inducing more interferences; consequently, for these clusters, we will relax or shrink the parameters in the filtering step according to the actual situation, and for those that do not have enough haplotype-tagged ratio, HapKled shrinks the parameters.

Formally, for a certain cluster, note the number of signatures from successfully haplotype-tagged reads as M , the total number of signatures as N , and the numbers of signatures from haplotype 1 and 2 as $H_{1}$ and $H_{2}$ , respectively. Define $H=\frac{M}{N}$ and $HR=\frac{\max\;\left(H_{1},H_{2}\right)}{M}$ . For parameters $H_{0},{HR}_{0},HomoR,HomoSF,HomoSR,LowHRF,LowHRR\in \left[0,1\right]$ , $NonHomoSF,NonHomoSR\in \left[-1,1\right]$ , if $H>H_{0}$ and $HR>{HR}_{0}$ , the filtering conditions for this cluster are multiplied by ${SC}_{Homo}=1-\left(HomoSF+HomoSR*\left(H-H_{0}\right)\right)$ . If $H>H_{0}$ and $HR>{HR}_{0}$ , the filtering conditions for this cluster are multiplied by ${SC}_{NonHomo}=1-\left(HomoSF+HomoSR*\left(H-H_{0}\right)\right)$ . If $H<H_{0}$ , the filtering conditions for this cluster are multiplied by ${SC}_{LowHR}=1+\left(LowHRF+LowHRR*\left(H_{0}-H\right)\right)$ .

### 2.3 Implementation of experiments

To evaluate the results of HapKled, we implemented experiments on simulated data and real ONT data of HG002. Along with HapKled (v1.0), we also tested four state-of-the-art tools, e.g., kled (v1.2.9), cuteSV (v2.1.0), Sniffles2 (v2.2), and Duet (v1.0), for comparison.

We simulated 24,919 SVs including 12,365 deletions, 12,176 insertions, 185 duplications, and 193 inversions, alongside SNVs from HG403 and generated indels, and added them to the GRCh38 reference using VISOR (v1.1.2) (Bolognini et al., 2020) to get two haplotypes of the simulated genome. We then used lrsim (v0.2) (https://github.com/CoREse/lrsim) to simulate ONT-like 30x long-read reads and subsequently aligned those reads to the GRCh38 reference using Minimap2 (v2.17-r941) (Li, 2018) to get the alignment file.

For real data, we obtained ONT ultra long sequencing data of the HG002 sample from https://ftp-trace.ncbi.nlm.nih.gov/ReferenceSamples/giab/data/AshkenazimTrio/HG002_NA24385_son/UCSC_Ultralong_OxfordNanopore_Promethion/HG002_GRCh37_ONT-UL_UCSC_20200508.phased.bam and downsampled it to 5×, 10×, 20×, and 30× using SAMtools (v1.19) (Danecek et al., 2021). To benchmark the results, we used GIAB HG002 SV v0.6 VCF and corresponding BED (Zook et al., 2020) as the gold standard.

All tested tools were run by default calling parameters, except cuteSV, which was run by applying “-s 2/3/4/5” for 5×/10×/20×/30× data. We used truvari (v4.1.0) (English et al., 2022) with parameters “-p 0.0” to benchmark the results. The calculations of precision, recall, and F1 are listed in Eqs 4–6:

$$precision=\frac{{TP}_{call}}{{TP}_{call}+{FP}_{call}},$$ (4)

$$recall=\frac{{TP}_{base}}{{TP}_{base}+{FN}_{base}},$$ (5)

$$F1=\frac{2\times precision\times recall}{precision+recall},$$ (6)

where ${TP}_{call}$ and ${FP}_{call}$ are the number of correct and incorrect detections, respectively, and ${TP}_{base}$ and ${FN}_{base}$ are the number of correctly covered and not covered records in the ground truth set, respectively. The precision, recall, and F1 are calculated on presence and genotyping metrics, denoted as presence precision, presence recall, presence F1, GT-precision, GT-recall, and GT-F1. All scripts for experiments, including the scripts to generate the simulated data, are available at https://github.com/CoREse/HapKled/experiments.

## 3 Results

### 3.1 Results on the simulated dataset

**FIGURE 2:**
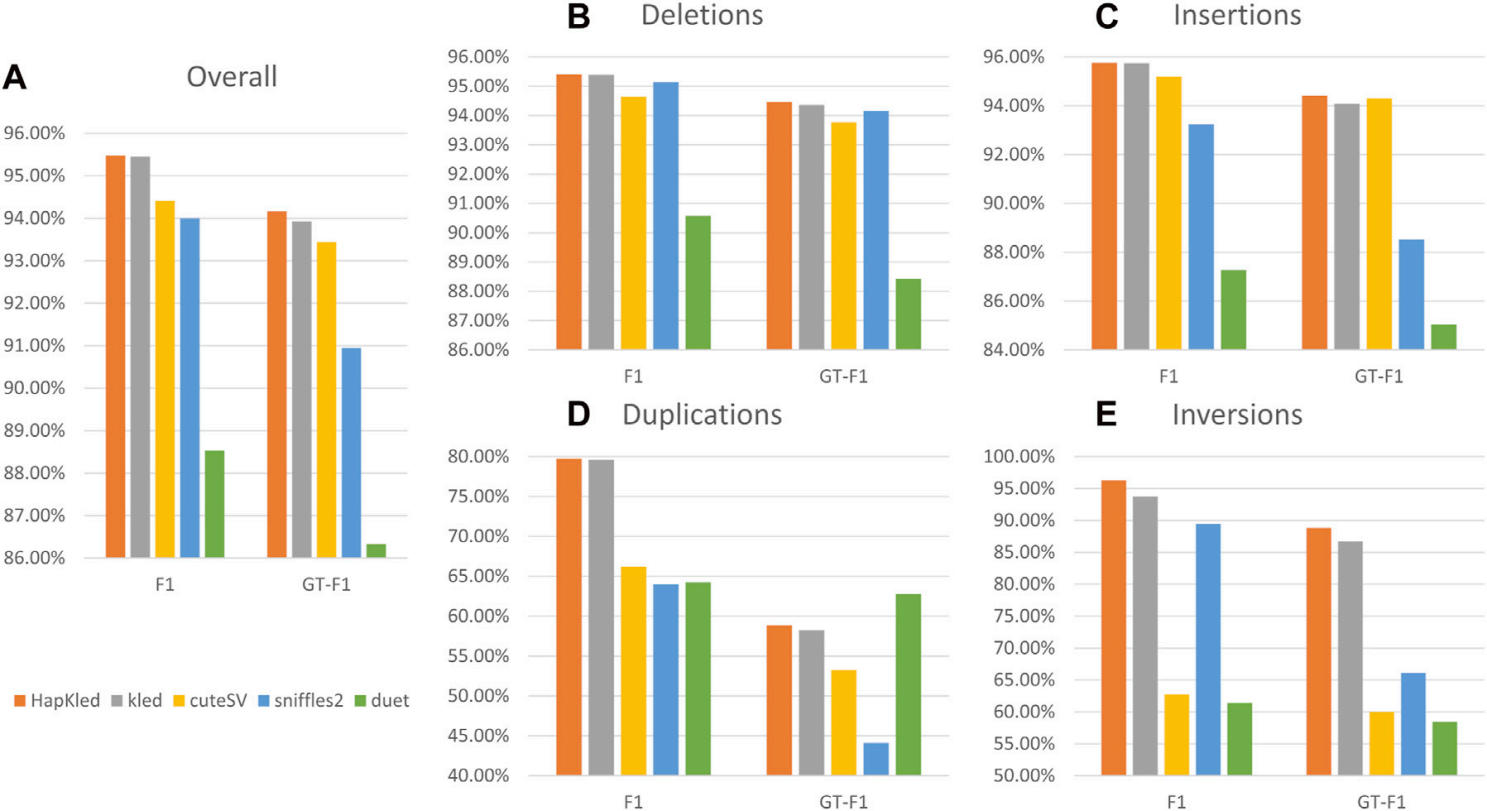
Benchmark experiment results on the simulated dataset. The vertical axes denote the F1 scores for presence or genotype. The subfigures include **(A)** the overall comparisons of presence F1 and GT-F1 of the tools and the comparisons of presence F1 and GT-F1 for **(B)** deletion, **(C)** insertion, **(D)** duplication, and **(E)** inversion.

To evaluate the performance of HapKled, we conducted an SV benchmark experiment on simulated 30x ONT-like data. Along with the HapKled, we also tested kled, cuteSV, Sniffles2, and Duet for comparison. In the benchmark experiment, HapKled achieved the best results in both presence F1 and GT-F1 in the overall experiment and the per-SV type experiments. For the overall experiment, HapKled achieved 95.48% presence F1 and 94.16% GT-F1, which are 0.03%–6.95% and 0.23%–7.83% higher than in other methods (Figure 2; Supplementary Table S1). As for the per-SV type benchmark, HapKled also obtained the highest presence F1s (95.4% for deletion, 95.76% for insertion, 79.75% for duplication, and 96.28% for inversion) and GT-F1s (94.46% for deletion, 94.42% for insertion, 58.86% for duplication, and 88.83% for inversion), which outperformed runner-up methods for each SV type by 0.01%–2.51% and 0.1%–2.11% (Figure 2; Supplementary Table S1). The fact that in all experiments HapKled achieved better performance compared to the original kled on both presence F1 and GT-F1 aspects validates the effectiveness of the haplotype-aware mechanics.

### 3.2 Results on real datasets of the HG002 sample

**FIGURE 3:**
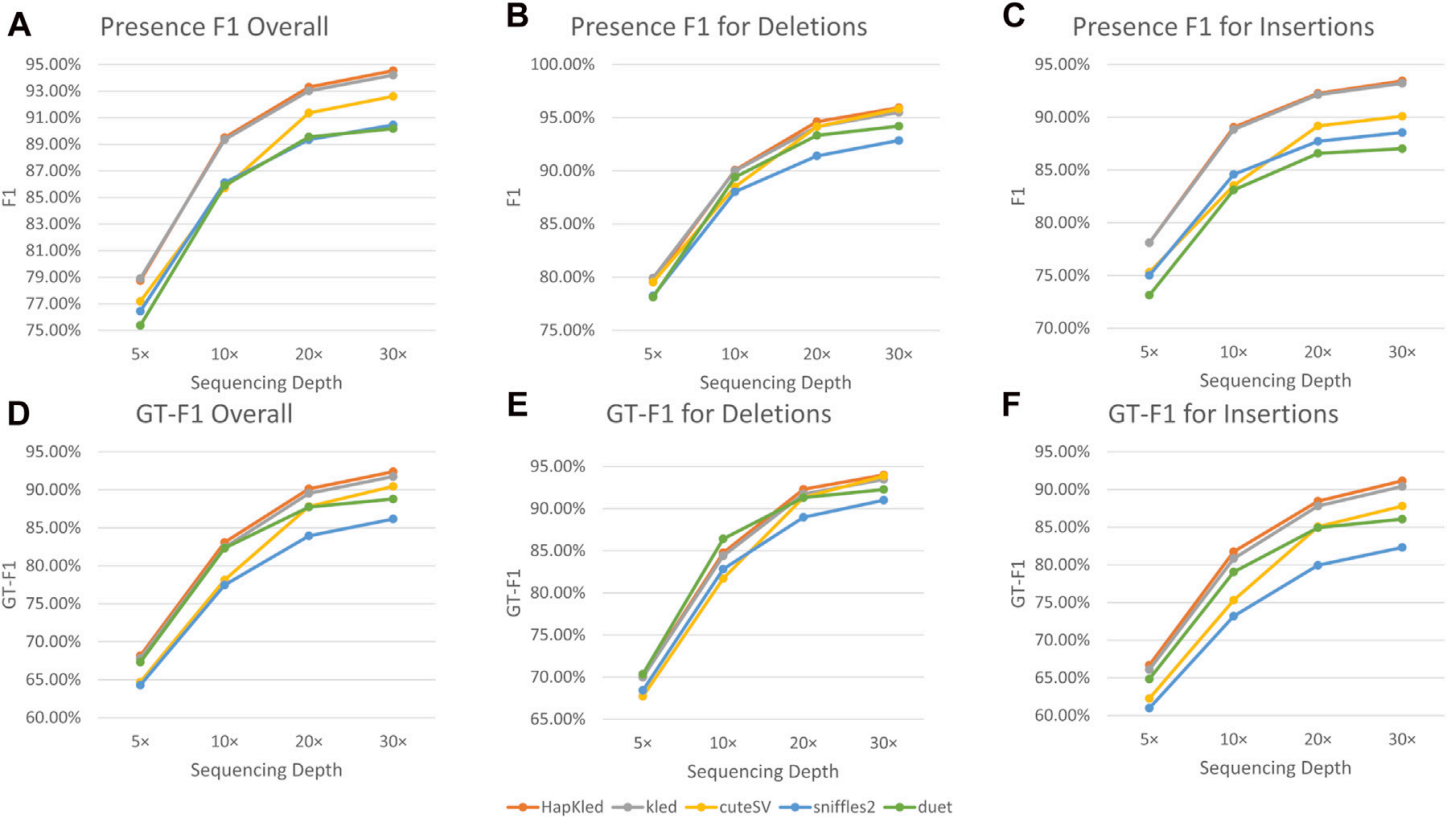
Benchmark experiment results on the HG002 ONT data. The vertical axes denote the F1 scores for presence or genotype. The subfigures include **(A)** the overall comparisons of presence F1; the comparisons of presence F1 for **(B)** deletion and **(C)** insertion; **(D)** the overall comparisons of GT-F1; the comparisons of GT-F1 for **(E)** deletion and **(F)** insertion.

Experiments performed on the simulated dataset proved the excellent SV detection capability of HapKled. To further test the real-world performance of HapKled, we conducted benchmark experiments on 30x HG002 ONT data, using the GIAB HG002 SV v0.6 VCF as the gold standard. Similar to the simulated experiments, in the real data experiments, HapKled delivered the best overall SV detection performance (presence F1: 94.54% and GT-F1: 92.39%), leading by 0.33%–4.36% for presence F1 and 0.64%–6.23% for GT-F1, compared to other tools (Figure 3; Supplementary Table S2). As for per-SV type performance, HapKled also managed to achieve the best presence F1 and GT-F1 for both deletion and insertion (presence F1: 95.94%, GT-F1: 93.99% for deletion and presence F1: 93.45%, GT-F1: 91.15% for insertion) and outperformed other tools by a minimum of 0.23% for presence F1 and 0.12% for GT-F1 (Figure 3; Supplementary Table S2).

To evaluate the performance of HapKled on lower sequencing depths, we down-sampled the real data to 5×, 10×, and 20×. In the 20× data, HapKled maintained the best presence F1s and GT-F1s (presence F1: 93.3%, GT-F1: 90.13% overall; presence F1: 94.62%, GT-F1: 92.29% for deletion; and presence F1: 92.27%, GT-F1: 88.45% for insertion), with 0.13–5.69% leads for presence F1 and 0.57–8.51% leads for GT-F1, compared to other tools (Figure 3; Supplementary Table S2). In the 5× and 10× data, HapKled continued to provide the highest F1s in most categories except for a few exceptions. In the 5× data, HapKled showed slightly lower overall presence F1 (78.74%) than vanilla kled (78.9%), and 1.56–3.36% higher than other tools (Figure 3; Supplementary Table S2). This is probably caused by the low haplotype-tagging quality of lower sequencing depth data. And in the 5× and 10× data, HapKled showed 0.31 and 1.65% lower GT-F1s for deletion compared to Duet, which also applies phase-aware mechanisms, but still delivered the best overall GT-F1s with 0.35–5.61% leads compared to other methods including Duet (Figure 3; Supplementary Table S2). The real data experiments conducted on HG002 ONT data show HapKled has the ability to solve real-world problems.

## 4 Discussion

In this article, we propose an SV detection tool HapKled, which utilizes the haplotype information underlying aligned sequencing data and delivers superior detection results.

Overall, HapKled can yield superior SV detection results compared to state-of-the-art tools, especially for genotyping performance. This is achieved by the combination of the extra haplotype information with the three haplotype-aware strategies applied in the calling processes. As shown in the Results section, HapKled delivered the best genotyping F1s across all tests on simulated and real data and best presence F1s on most tests, except for 5× data on HG002. Compared to the vanilla kled, HapKled shows a clear improvement, especially on genotyping performance, making the efforts of the haplotype-aware strategies evident.

While HapKled can achieve superior SV detection performance, its time consumption is significantly increased as well due to the introduction of the haplotype-tagging procedures, which include SNV detection and haplotype-tagging, both of which are time-consuming tasks. In other words, the haplotype-tagging procedures come with a price: they consume substantial time to improve the final SV detection. Nevertheless, we believe the price is worthwhile under many circumstances because the bottleneck of SV detection is usually not limited by the analysis speed, but by the accuracy and recall of the detection results; thus, sacrificing acceptable time for more reliable results is reasonable; furthermore, haplotype information can not only be used by SV detection during the whole procedure of genetic analysis, but it might be required by other analysis purposes, for example, *de novo* assembly of genomes; thus, in these projects, HapKled actually does not induce extra effort since the haplotype-tagged BAM file can be used in other procedures.

## Data Availability

Publicly available datasets were analyzed in this study. This data can be found here: https://ftp-trace.ncbi.nlm.nih.gov/ReferenceSamples/giab/data/AshkenazimTrio/HG002_NA24385_son/UCSC_Ultralong_OxfordNanopore_Promethion/, https://ftp-trace.ncbi.nlm.nih.gov/ReferenceSamples/giab/release/AshkenazimTrio/HG002_NA24385_son/NIST_SV_v0.6/. The scripts used to generate the simulated data can be found here: https://github.com/CoREse/HapKled/experiments.
